# Hantavirus Pulmonary Syndrome—The 25th Anniversary of the Four Corners Outbreak

**DOI:** 10.3201/eid2411.180381

**Published:** 2018-11

**Authors:** Charles J. Van Hook

**Affiliations:** Longmont United Hospital, Longmont, Colorado, USA

**Keywords:** hantavirus, Sin Nombre virus, viruses, hantavirus pulmonary syndrome, HPS, hemorrhagic fever with renal syndrome, HFRS, respiratory infections, outbreak, hemoconcentration, extracorporeal membrane oxygenation, ECMO, zoonoses, Four Corners Outbreak, Four Corners Region, southwestern United States, United States

## Abstract

During the spring of 1993, a mysterious respiratory disease struck the Four Corners region of the southwestern United States. Persons who became ill were generally young and previously healthy before succumbing to an acute febrile illness that began with simple influenza-like symptoms and often culminated in death by pulmonary edema and cardiovascular collapse. With astonishing speed and efficiency, a collaborative team of federal, state, and local healthcare workers, including clinicians, epidemiologists, and laboratory scientists, identified a newly discovered species of hantavirus as the causative agent of the outbreak. In the ensuing 25 years, the epidemiology, virology, pathophysiology, clinical course, and treatment of hantavirus pulmonary syndrome have been the focus of ongoing research. Because of its rarity, and because of the need for early acute intervention in the face of precipitous decline, recognition of the unique laboratory profile of hantavirus pulmonary syndrome in the setting of a predisposing exposure history is of paramount importance.

On the morning of May 14, 1993, a 19-year-old Native American man was traveling by car through the Four Corners region of New Mexico, USA—the area where New Mexico, Arizona, Colorado, and Utah meet—when he became so severely short of breath that his alarmed accompanying family members pulled into a nearby service station to call for help. By all measures, the young man, a competitive marathon runner of local renown, had been in previous good health. A few days earlier, he had visited an outpatient clinic because of fever and myalgia, was treated symptomatically, and was well enough early on the morning of May 14 to embark on a trip from his home in Crownpoint, New Mexico, to Gallup, New Mexico. However, by the time the responding ambulance crew arrived, he had collapsed because of respiratory failure. He was taken to the emergency department at the Gallup Indian Medical Center, where he found to have florid pulmonary edema, and where, despite maximal resuscitative efforts, he died in the emergency department.

The emergency department medical staff was understandably bewildered as to why an extremely fit adolescent athlete would so swiftly die from acute pulmonary edema. In New Mexico, any unexplained, suspicious, or otherwise irregular death is, by law, reportable to the New Mexico Office of the Medical Investigator. The officer on duty that day in Gallup was a young investigator named Richard Malone.

After arriving at the hospital and hearing the clinical narrative, Malone was startled by the resemblance of this death to another death that he had investigated a few weeks earlier at the same facility. At that time, he had been called after a young woman, also a Navajo tribal member, had died from acute pulmonary edema without any clinical clues pointing to a distinct etiology. Malone had referred that case for a postmortem examination to Patricia McFeeley, a University of New Mexico pathologist who worked in conjunction with the office of the medical examiner. McFeeley had reported that the young woman had died from pulmonary edema that was evident by gross and microscopic examinations. The heart of this patient was structurally normal, and results of serologic and microbiologic tests were nonrevealing.

The pathologist was admittedly puzzled by the case and had discussed her uneasiness with Malone. McFeeley was again at work in Albuquerque on the morning of May 14, and when Malone called and shared his thoughts on the similarity of the 2 cases, she readily agreed to perform an autopsy on the deceased person. With that, Malone made his way toward the emergency department waiting room to approach the family of the young man about obtaining permission to transport the body to the state laboratory in Albuquerque. Mr. Malone expected that he would have to gently persuade the family to agree, because the Navajo people are generally resistant to any action that could be perceived as disturbing the newly dead. When he met the gathered family, he was shocked by their shared story.

The patient had been en route to Gallup from his home in the small Navajo reservation village of Crownpoint that morning to attend a funeral, which was about to begin at a mortuary literally across the street from the Indian Medical Center. The planned funeral was that of his fiancée, the 21-year-old mother of his infant child. The young woman, who was also an active runner, had died only days earlier at an outlying rural reservation clinic. She had also complained only of antecedent fever and myalgia, and the decline in her health had been so precipitous at the remote clinic that there had been inadequate time to transport her to a fully staffed facility. Because Crownpoint is located on the Navajo reservation and governed by tribal rather than state law, the clinic there was not required to adhere to reporting requirements of New Mexico. Consequently, Malone’s office had no record of her death or of the surrounding circumstances. Malone recognized the relevance of this small cluster of cases, and after quickly updating McFeeley by telephone, he convinced the family of the young woman to allow her remains to be examined in Albuquerque. Malone invoked the health of their surviving infant child as a deciding factor in convincing reluctant family members to allow the state to proceed with their autopsies.

After ensuring that both bodies had been secured for transport, Malone sought out Bruce Tempest, the physician who served as the medical director for the Gallup Indian Medical Center. While he listened to Malone’s report, Tempest remembered that he had been involved in at least 2 recent informal consultations with other physicians who had cared for young, previously healthy tribal members who had died in a dramatic fashion from a mysterious respiratory illness. Both men agreed that immediate further action was mandated. They decided that Malone would scour the records of the state coroner for information, and that Tempest would survey his clinical colleagues in the Four Corners area for similar cases.

The postmortem examinations of the 2 new case-patients showed only unexplained, severe pulmonary edema. Malone and Tempest quickly uncovered several new suspicious cases from the preceding few months, and on May 17, 1993, the New Mexico Department of Health was notified of their concerns. The state officials crafted a letter that was sent to clinicians in the 4-state area of Arizona, Colorado, New Mexico, and Utah. The communication offered a brief description of the cases to date and asked that any similar cases be reported immediately to them. The mailing was effective in identifying several other potential cases.

Unfortunately, soon thereafter, when the lay press reported that an unexplained illness was killing young tribal members throughout the Four Corners region, a near panic of the general populace ensued. Navajo and Hopi people were shunned, disinvited from regional athletic events, and made to feel unwelcome in public places. Politicians were pressured to act. On May 28, the Friday afternoon of Memorial Day weekend, New Mexico state health officials contacted the Centers for Disease Control and Prevention (CDC), described their predicament, and asked for expert assistance.

Within hours of the call for help, a team of investigators assembled and mobilized. Jay Butler, an experienced epidemiologist in the Epidemic Intelligence Service at CDC, was designated as the leader. Two young Epidemic Intelligence Service officers (Ronald Moolenar and Jeffrey Duchin) assisted him. Less than 24 hours after the group had been organized, they arrived at the Albuquerque airport and were shuttled to the campus of the University of New Mexico, where they were joined by members of the University of New Mexico medical faculty, Indian Health Service physicians, and various other state and federal health officials.

The first order of business was case definition, and the health officials agreed to evaluate any patient from the area who, going forward from January 1, 1993, had demonstrated imaging evidence of unexplained bilateral infiltrates with associated hypoxemia. The team would also evaluate any death that had occurred with unexplained pulmonary edema. More than 30 suspected cases, with varying degrees of available clinical information, were presented to the group. The assembly then evolved into a brainstorming session, where participants were invited to offer their thoughts about potential etiologies of the outbreak. Various ideas were put forth, ranging from the exotic to the mundane. Plague, tularemia, anthrax, and multiple other potential diseases were dismissed as possibilities because of a lack of any corroborating evidence.

By the end of the long weekend, the consensus was that the outbreak was the result of 1 of 3 possible causes. The first consideration was that of a new, aggressive, and previously unrecognized type of viral influenza. The second was that an environmental toxin was the causative agent, which was certainly plausible in an agricultural area with a less than optimal regulatory climate and a history of military weapons testing. The third listed possibility was the most fascinating: that a previously unrecognized pathogen was the cause of the epidemic (*1*).

On Tuesday, June 1, fifteen members of the CDC team began an on-site, meticulous, review of medical records. They also procured tissue specimens from suspected cases, which were flown to CDC headquarters in Atlanta, Georgia, for immediate analysis. Epidemiologists interviewed patient and control families and performed detailed inspections of their homes and workplaces.

By Friday, June 4, scientists of the Special Pathogens Branch at CDC had tested extracted IgM from 9 patients with a panel of 25 different virus stock samples from the laboratory at CDC. Antibody from all 9 patients showed cross-reactivity with each of 3 different hantavirus species and with none of the other 22 viruses. Hantaviruses were known to be the causative agents of a family of diseases of varying severity, collectively known as hemorrhagic fever with renal syndrome (HFRS), which affect patients in the Northern Hemisphere from Scandinavia to the Korean Peninsula. The 3 hantavirus samples initially tested were Hantaan virus, the cause of Korean hemorrhagic fever; Seoul virus, the causative agent of a form of HFRS common in Asia; and Puumala virus, the cause of a relatively mild form of HFRS in northern Europe. Shortly thereafter, the same samples were found to cross-react with Prospect Hill virus, which was known to infect voles in Maryland but had never been isolated from human tissue or associated with human disease (*2*).

Several members of the investigating team had extensive international infectious disease experience and knowledge of the epidemiology and clinical course of HFRS. The illness was known to be caused by different types of hantavirus and to be transmitted to humans by inhalation of virus shed in rodent droppings. The syndrome is characterized by an enormous change in vascular endothelial permeability, predominantly in the kidney, with the loss of massive amounts of intravascular fluid into the renal extravascular parenchyma and retroperitoneal space. The degree of intravascular fluid depletion is so severe that hemoconcentration occurs, and patients often have pronounced increases in hemoglobin concentrations and hematocrit values.

The clinicians of the investigation team had noted high levels of hemoconcentration in several of the potential cases and, in light of the CDC findings, they suspected that they were now dealing with a new hantavirus disease. This conclusion was a substantial leap of thought for several reasons. At the time, in the Western Hemisphere, hantaviruses were recognized as infecting only rodents, and no case of human disease had been described. In addition, the study group patients had little evidence of renal involvement; the predominant target organ was the lung in all cases. Undeterred by these discrepancies, some group members postulated, in a profoundly prescient fashion, that the outbreak was caused by an as-yet-unrecognized hantavirus that targeted the pulmonary capillary endothelium.

Acting upon the new information, CDC dispatched a rodent trapping team to New Mexico. Over the ensuing week, ≈1,700 rodents were captured at patient and control sites. The most commonly secured rodent was *Peromyscus maniculatus*, the deer mouse (*3*).

Concurrently, the Special Pathogens Branch in Atlanta worked feverishly to uncover the new hantavirus. On June 10, using reverse transcription PCR technology, these scientists were able to obtain a sequence from the medium segment of the RNA strand of the suspected virus. The Viral Pathology Laboratory also identified hantaviral antigens in endothelium of the pulmonary capillary bed and other tissues (*4*). Less than 1 week later, on June 16, the same team identified an identical virus basepair sequence, as well as a prevalence of hantavirus antibody, from *Peromyscus maniculatus* mouse specimens trapped on site (*5*). The virus and its rodent reservoir had been definitively identified less than 3 weeks after CDC had assembled its task force.

The new virus proved difficult to culture, and it was not until November 1993 that teams from the CDC and the US Army Medical Research Institute of Infectious Diseases (Fort Detrick, MD, USA) were able to culture the virus. Their initial recommendation was to name the pathogen Muerto Canyon virus, after an involved area on the Navajo Reservation. The Navajo people reacted strongly against any further association with the disease that had led to so much initial prejudice, and tribal elders appealed to officials to reconsider. Ultimately, the new agent was officially named Sin Nombre virus (virus with no name).

While the bench scientists were successfully identifying the pathogen, epidemiologists and clinicians were clarifying the clinical course of the newly recognized syndrome. Eighteen patients were found to have had either serologic or PCR evidence of infection. These patients were mostly young adults, with a noticeable sparing of the extremes of life. Physical examinations were remarkable for fever, tachypnea, tachycardia, and hypotension. Severe pulmonary edema was nearly ubiquitous, and the mortality rate in the initial outbreak exceeded 75%. A distinct laboratory pattern was prominent, characterized by hypoxemia, leukocytosis with the presence of peripheral immunoblasts, hemoconcentration with a marked increase in hemoglobin and hematocrit, thrombocytopenia, and increased prothrombin and partial thromboplastin times. The predominant chest radiograph finding was bilateral parenchymal infiltrates. An unusual hemodynamic profile was also observed. Patients in whom pulmonary artery catheters had been placed showed a severe reduction in cardiac output and a marked increase in systemic vascular resistance, in association with normal or low pulmonary capillary wedge pressures, consistent with cardiogenic shock and noncardiogenic pulmonary edema. Histopathologic examination of the lungs of patients who died showed moderate lymphoid interstitial infiltration with severe alveolar edema (*4*).

The remarkable work of the Hantavirus Study Group, describing the newly defined hantavirus pulmonary syndrome (HPS), was published in the April 7, 1994, edition of The New England Journal of Medicine (*6*). A glowing editorial reviewing the team effort appeared in the same issue.

A burning question for the scientific community remained. Why did the outbreak occur in the Four Corners Region, and why did it happen in the spring of 1993? Biologists from the University of New Mexico happened to be studying the deer mouse population in that region at that time. They found that the mouse population in 1993 was 10-fold greater than it had been during the preceding spring. Working with a team of environmental scientists, those biologists demonstrated that, because of the increased moisture of an El Niño winter, there was a relative abundance of springtime vegetation in the Four Corners region that provided shelter and food for regional fauna. The resultant explosive growth of the rodent population was followed by increased human exposure to the deer mouse vector (*7*).

## HPS Today

In the ensuing 25 years, the epidemiology, virology, pathophysiology, clinical course, and treatment for HPS have been the focus of ongoing research. The Sin Nombre virus is a single-stranded, 3-segmented RNA virus of the family *Hantaviridae.* It is the cause of chronic, seemingly innocuous, and persistent infections in the host rodent. Like all hantaviruses, the Sin Nombre virus is principally associated with only 1 rodent species, in this case, the common deer mouse. Other rare hantavirus species in North America have been associated with variants of HPS, and they are also associated predominantly with 1 rodent species.

The disease remains extremely uncommon: <800 HPS cases have been reported in the United States during the past 25 years (*8*). There is a western states predominance of this disease, and almost all cases are caused by exposure at home or in the workplace (*9*). The temporal and spatial distribution of cases reflect fluctuations in the population of the rodent host as the virus is transmitted to humans by inhalation of aerosolized particles shed in rodent excreta. Human-to-human spread has never been reported in North America.

The virus has a remarkable predilection for pulmonary capillary endothelial cells and a complex and still poorly understood pathogenesis. Infection by inhalation is followed by an incubation period of 1–5 weeks. A 3–6-day prodromal period then occurs, during which patients first exhibit fever, with respiratory symptoms notably absent. The development of a cough signals the onset of the fulminant cardiopulmonary phase, which is characterized by severe capillary leak, extraordinary pulmonary edema, and myocardial depression, and lasts for up to 1 week. The current mortality rate during the cardiopulmonary phase is ≈40%. Survivors mobilize third-space fluid during the diuretic-recovery phase, which may last up to 2 weeks (*10*).

Most of the damage during the cardiopulmonary phase of HPS is directly related to cell-mediated immunity gone awry. During the incubation phase, there is a ubiquitous deposition of the virus within the pulmonary endothelium, with no associated changes in either the structural integrity or permeability of the microenvironment (*4*). During the relatively brief prodromal phase, circulating immunoblasts appear and humoral antibody is produced. It is during the cardiopulmonary phase that well-differentiated T cells appear on site and participate in the release of soluble mediators (among which tumor necrosis factor-α is prominent); massive changes in pulmonary capillary endothelial cell permeability result (*11*). Fluid loss into the alveolar and pleural spaces is so voluminous that the heart becomes preload deprived and cardiac output decreases. The same soluble mediators are in part responsible for depression of myocardial contractility that often leads to frank cardiovascular collapse (*12*).

A distinctive hematologic laboratory profile offers clues to the diagnosis of HPS. Researchers at the University of New Mexico have triaged patients with prodromal symptoms and a consistent exposure history by examining the peripheral blood smear for the presence of 5 factors: thrombocytopenia, hemoconcentration, a granulocytic left shift, the absence of toxic change, and the presence of >10% immunoblasts. If 4 of the 5 factors are present, there is ≈a 90% sensitivity and specificity for the disease (*13*). Definitive diagnosis of hantavirus infection relies on serologic testing for IgM and IgG, which is highly sensitive and specific, but because of travel times to laboratories performing the assay, it takes >72 hours in most cases to provide results.

Treatment of HPS is challenging. Because of the severity of endothelial leakage, fluid resuscitation can lead to worsening pulmonary edema. Because patients are maximally vasoconstricted, vasopressors are of little benefit. Inotropes, particularly dobutamine, are often used but have no demonstrated value. Steroids have been used, again with no proven benefit (*14*). Patients in the cardiopulmonary phase have a failing heart and failing lungs. Extracorporeal membrane oxygenation (ECMO) offers a supporting bridge to the diuretic-recovery phase. The University of New Mexico has pioneered the use of ECMO for HPS. Early efforts there to use ECMO were plagued by technical difficulty because health of HPS patients has a tendency to decline so rapidly that clinicians were often left attempting to obtain arterial and venous ECMO access for patients in full cardiac arrest (*15*). Physicians at the University of New Mexico have now developed a strategy of preemptively placing femoral arterial and venous access catheters in suspected case-patients at the time of hospital arrival, so that ECMO can be initiated at the earliest sign of decompensation. This strategy has resulted in an impressive 80% survival rate in patients despite overt cardiopulmonary collapse. Mortality rates in that subset of patients had previously exceeded 90% (*16*).

HPS was discovered and defined by the collaborative efforts of federal, state, and local investigators during the spring of 1993. The rapidity of the successful investigation was the result of the presence of competent persons at all levels and to the early actions of the New Mexico Office of Medical Investigation. HPS is a disease of healthy young persons who have a history of rodent exposure, usually at home or at work. A laboratory examination that demonstrates hemoconcentration, or the presence of immunoblasts on a peripheral blood smear, should raise immediate clinical suspicion. Severe capillary leak with massive pulmonary edema is the hallmark finding of HPS. Frederick Koster, an infectious disease physician in New Mexico who was part of the original Four Corners investigation, has described HPS associated pulmonary edema as “a disease manifestation without parallel in clinical medicine” (*17*). Suspected case-patients should always be referred to an ECMO-capable center without delay because health decline is precipitous, and ECMO may be lifesaving.
